# Studies on the mother flower carnation: past, present, and future

**DOI:** 10.1093/hr/uhaf118

**Published:** 2025-04-29

**Authors:** Min Wang, Zhengkang Pi, Zekang Pan, Xilin Li, Linlin Zhong, Yunjiang Cheng, Manzhu Bao, Fan Zhang

**Affiliations:** National Key Laboratory for Germplasm Innovation & Utilization of Horticultural Crops, Huazhong Agricultural University, Wuhan 430070, China; Hubei Hongshan Laboratory, Wuhan 430070, China; National R&D Center for Citrus Postharvest Technology, Huazhong Agricultural University, Wuhan 430070, China; Joint International Research Laboratory of Germplasm Innovation & Utilization of Horticultural Crops, Huazhong Agricultural University, Wuhan 430070, China; National Key Laboratory for Germplasm Innovation & Utilization of Horticultural Crops, Huazhong Agricultural University, Wuhan 430070, China; Hubei Hongshan Laboratory, Wuhan 430070, China; National R&D Center for Citrus Postharvest Technology, Huazhong Agricultural University, Wuhan 430070, China; Joint International Research Laboratory of Germplasm Innovation & Utilization of Horticultural Crops, Huazhong Agricultural University, Wuhan 430070, China; National Key Laboratory for Germplasm Innovation & Utilization of Horticultural Crops, Huazhong Agricultural University, Wuhan 430070, China; Hubei Hongshan Laboratory, Wuhan 430070, China; National R&D Center for Citrus Postharvest Technology, Huazhong Agricultural University, Wuhan 430070, China; Joint International Research Laboratory of Germplasm Innovation & Utilization of Horticultural Crops, Huazhong Agricultural University, Wuhan 430070, China; National Key Laboratory for Germplasm Innovation & Utilization of Horticultural Crops, Huazhong Agricultural University, Wuhan 430070, China; Hubei Hongshan Laboratory, Wuhan 430070, China; National R&D Center for Citrus Postharvest Technology, Huazhong Agricultural University, Wuhan 430070, China; Joint International Research Laboratory of Germplasm Innovation & Utilization of Horticultural Crops, Huazhong Agricultural University, Wuhan 430070, China; National Key Laboratory for Germplasm Innovation & Utilization of Horticultural Crops, Huazhong Agricultural University, Wuhan 430070, China; Hubei Hongshan Laboratory, Wuhan 430070, China; National R&D Center for Citrus Postharvest Technology, Huazhong Agricultural University, Wuhan 430070, China; Joint International Research Laboratory of Germplasm Innovation & Utilization of Horticultural Crops, Huazhong Agricultural University, Wuhan 430070, China; National Key Laboratory for Germplasm Innovation & Utilization of Horticultural Crops, Huazhong Agricultural University, Wuhan 430070, China; Hubei Hongshan Laboratory, Wuhan 430070, China; National R&D Center for Citrus Postharvest Technology, Huazhong Agricultural University, Wuhan 430070, China; Joint International Research Laboratory of Germplasm Innovation & Utilization of Horticultural Crops, Huazhong Agricultural University, Wuhan 430070, China; National Key Laboratory for Germplasm Innovation & Utilization of Horticultural Crops, Huazhong Agricultural University, Wuhan 430070, China; Joint International Research Laboratory of Germplasm Innovation & Utilization of Horticultural Crops, Huazhong Agricultural University, Wuhan 430070, China; The Institute of Flowers Research, Huazhong Agricultural University, Wuhan 430070, China; Key Laboratory of Huazhong Urban Agriculture, Ministry of Agriculture and Rural Affairs, Huazhong Agricultural University, Wuhan 430070, China; National Key Laboratory for Germplasm Innovation & Utilization of Horticultural Crops, Huazhong Agricultural University, Wuhan 430070, China; Hubei Hongshan Laboratory, Wuhan 430070, China; National R&D Center for Citrus Postharvest Technology, Huazhong Agricultural University, Wuhan 430070, China; Joint International Research Laboratory of Germplasm Innovation & Utilization of Horticultural Crops, Huazhong Agricultural University, Wuhan 430070, China; The Institute of Flowers Research, Huazhong Agricultural University, Wuhan 430070, China; Key Laboratory of Huazhong Urban Agriculture, Ministry of Agriculture and Rural Affairs, Huazhong Agricultural University, Wuhan 430070, China; Yunnan Seed Laboratory, Kunming 650200, China

## Abstract

Carnation (*Dianthus caryophyllus* L.) is an important global flower crop, with great ornamental and economic value. It has more than 2000 years of cultivation history and profound cultural heritage known as mother flower. Now, although carnation is deeply loved by the majority of consumers because of its rich color and various varieties, the original carnation unique clove flavor has disappeared. Furthermore, our understanding of carnation traits such as flower shape, flower color, flower fragrance, disease resistance, and vase life remains limited. Previous reviews have primarily concentrated on individual aspects of carnation, failing to present a comprehensive overview. In this review, we summarize the recent progress of carnation in these aspects, so as to provide a reference for the future research direction in carnation.

## Introduction

Carnation (*Dianthus caryophyllus* L.) is one of the most important flowers in the world, which has high ornamental and economic value. It is cultivated year-round in the temperate regions, especially in the cool highlands of Colombia, Kenya, and China [1]. Carnation originated in the southern Europe, the Mediterranean, France to Greece area, has more than two thousand years of cultivation history. The Greek and southern European coasts of Athens cultivated large numbers of carnations and revered them as ‘Dianthus’ derives from the Greek words ‘Dios’ and ‘Anthos’, meaning the flower of Zeuss. In addition, ancient Greeks used carnation to make garlands and crowns for gods, which is also a symbol of glory, so carnation is also called the coronation flower [2].

As one of the world’s most significant and popular cut flowers, the planting area of carnation reached more than 149 million hectares, ranking first in the world in 2022. Carnation can be used not only for cut flowers, but also for borders, edging, bedding purposes, pots and rock gardens. However, after long-term breeding, although modern carnations have richer flower colors and longer flowering periods, and their disease resistance is better, their original fragrance is fading or disappearing. In addition, the understanding of carnation’s flower color, flower fragrance, flower pattern, and other aspects is still limited. So, in this review, we summarize the research progress of carnation in various aspects, in order to provide reference value for the follow-up carnation breeding.

## The carnation germplasm resources

**Figure 1:**
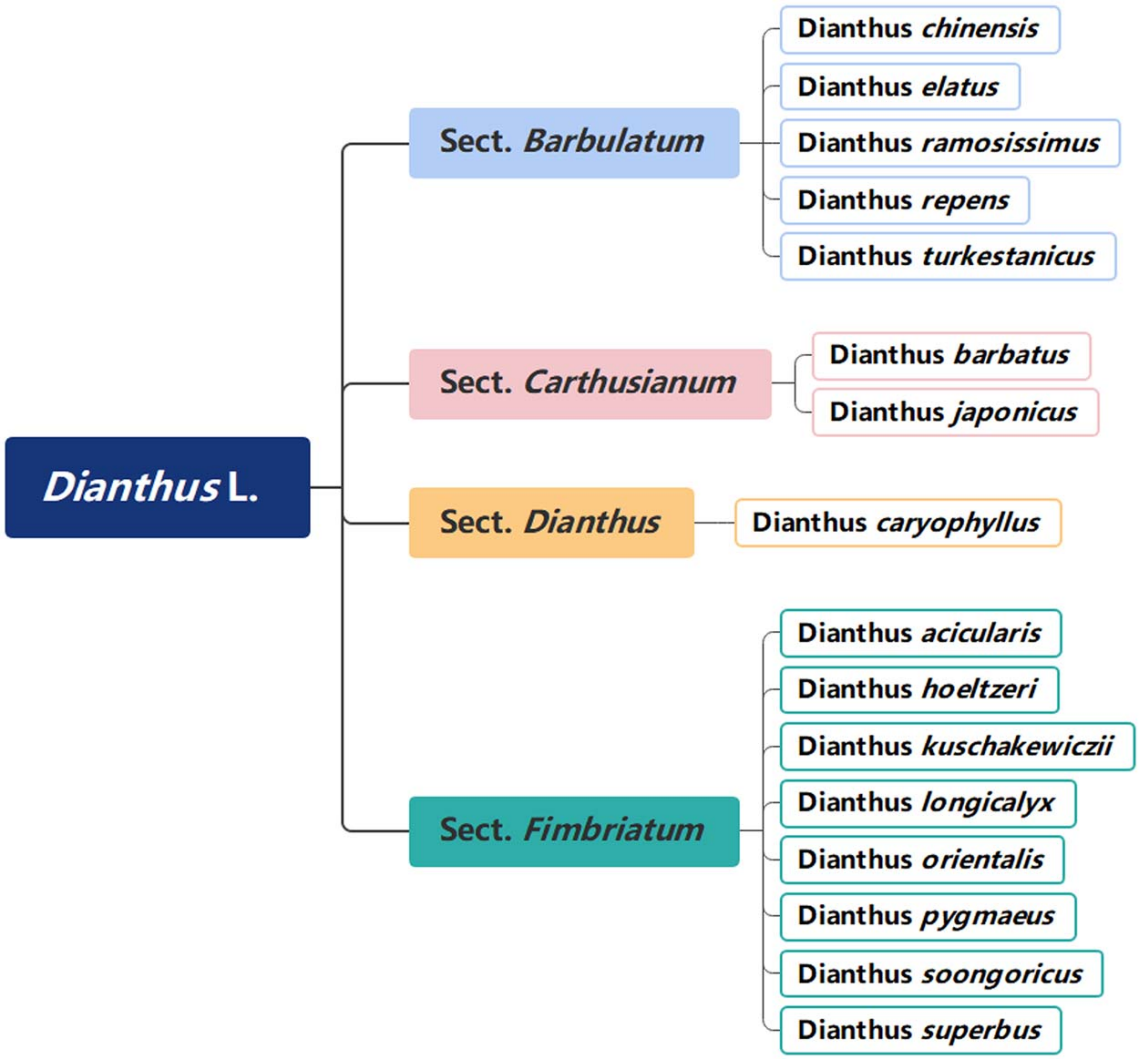
Classification of *Dianthus* L. wild resources in China.

The family Caryophyllaceae includes more than 80 genera and 3000 species and is predominantly distributed throughout the arctic (i.e. temperate to arctic parts of Eurasia and North) [3]. The genus *Dianthus* has about 600 species of annual, biennial, and perennial herbs, and *Dianthus caryophyllus* is the most important species commercially. The genus *Dianthus* widespread in the northern temperate zone, most of in the Mediterranean region of Europe and Asia, a few in America and Africa. There are 16 species and 10 varieties in China, most of which are distributed in northern grasslands and mountainous grasslands [4] (Fig. 1).

**Figure 2:**
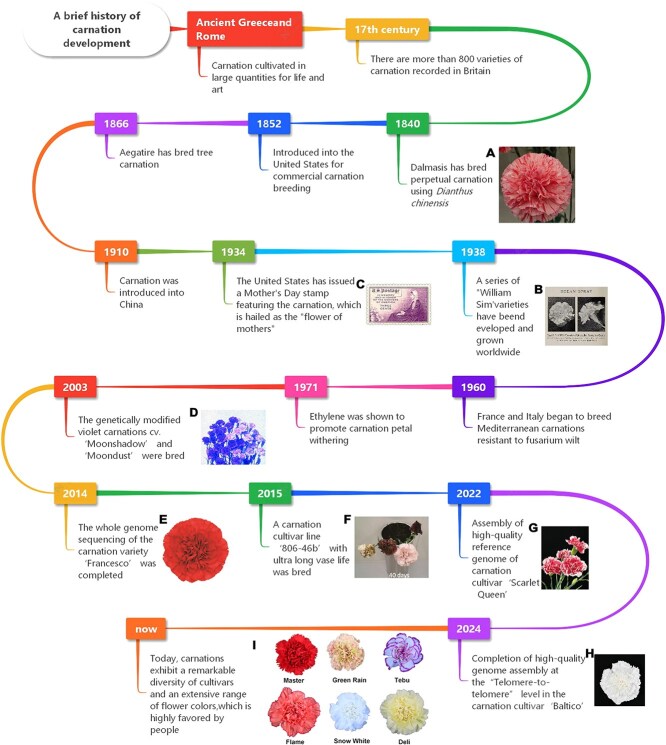
The brief history of carnation development. Panels A, B, C, and E are from the Internet; Panels D, F, G, and H are from the corresponding literature cited in the text.

Variety improvement of carnation began in the 16th century, mainly in European gardens. The first modern carnation to bloom all year round was the ‘Ativn’, which was developed by the frenchman Dalmais between 1835 and 1845. In 1852, carnation was introduced into the United States and became the parent of many fine American varieties. Hundreds of commercial carnation varieties were developed, such as ‘William Sim’, bred by William Sim in 1938, is a famous standard carnation. But it is highly susceptibility to fusarium wilt and bacterial wilt. In 1960 or so, France and Italy have started the cultivation of ‘Mediterranean’ type antiwithering carnations. The ‘Mediterranean’ carnation has excellent flower shape and high flower quality, which reduces or eliminates the calyx dehiscence of ‘Sim’ type varieties. At present ‘Sim’ type varieties have disappeared, most of the standard commercial varieties are ‘Mediterranean’ type (Fig. 2).

## Unraveling the mystery of carnations’ genome

With the rapid development of sequencing technology and assembly algorithms in recent years, genomics has become a powerful tool for uncovering the mysteries of plant genes [5–12]. Plant genome research extends beyond decoding genetic information, it also plays a crucial role in ecology, evolution, agriculture, and molecular breeding, offering unprecedented opportunities for understanding and application [13]. More than 300 *Dianthus* species were produced whereas many of their genomes remain to be sequenced [14]. This highlights the importance of applying genomic technology to carnation research.

Most carnation cultivars are diploid, with a chromosome number of 2*n* = 2*x* = 30. Several carnation genomes have been assembled using different sequencing technologies. The key features of these genomes are summarized in Table 1. The first carnation genome of *D. caryophyllus* cv. ‘Francesco’ was released in 2014, generated using only Illumina and GS FLX+ short-read technologies. A total of 45 088 scaffolds were obtained, covering 91% of the estimated carnation genome size based on k-mer analysis. At the same time, the researchers constructed the first carnation genome database carnation DB (https://carnation.kazusa.or.jp/) [15]. However, next-generation sequencing (NGS) has the disadvantage of short read-lengths, and genome assembly is at the scaffold level, which introduces more errors when facing complex assemblies such as highly repetitive genomes. With further innovations in technology, we are now in the era of third-generation sequencing (TGS), which is characterized by its ability to produce very long reads, overcoming the short-read drawbacks of NGS. In 2022, the researchers assembled the genome of *D. caryophyllus* ‘Scarlet Queen’ (a well-known cultivar with petal margin coloration) based on sequence data generated from the PromethION platform by Oxford Nanopore Technologies (ONT) and constructed pseudo-chromosomes using high-throughput chromosome conformation capture (Hi-C) techniques [16]. This study assembled the carnation genome at the chromosome level for the first time. By integrating transcriptomic and metabolomic analyses, it investigated the molecular mechanisms underlying key ornamental traits, including flower color, shape, and scent. Key genes involved in these traits were identified, providing a foundation for the targeted improvement of ornamental characteristics and the breeding of new carnation varieties. However, chromosome-level genome assembly still contains numerous gaps and assembly errors. Researchers assembled the chromosome-scale, haplotype-resolved genome of *D. caryophyllus* ‘Aili’. Utilizing PacBio high-fidelity (HiFi) long reads and Hi-C technologies, they generated genome assemblies of 584.88 Mb (hap1) and 578.78 Mb (hap2) and identified significant structural variations between haplotypes. Phylogenetic analysis indicated that carnation underwent a whole-genome triplication (WGT) event. This high-quality genome assembly serves as a valuable resource for molecular breeding, evolutionary studies, and functional genomics research [14]. In 2023, researchers successfully assembled the telomere to telomere (T2T) carnation genome of the variety ‘Baltico’ [17]. The T2T genome assembled in this study provides new insights into the analysis of telomere and centromere regions, enabling the identification of specific centromeric features that could not be detected through high-order repeats in carnations. Researchers analyzed allele-specific expression in three different tissues. The results indicate that gene length, coding sequence, intron size, exon number, and transposable element insertion correlate with gene expression levels and ratios.

**Table 1:** Overview of carnation genome assemblies

	Level	Genome size	Contig N50	Sequencing technology
‘Francesco’	Scaffold	568.89 Mb	17.55 Kb	Sanger sequencing GS FLX+
‘Scarlet Queen’	Chromosome	636.30 Mb	14.67 Mb	ONT Illumina-short Illumina-HiC
‘Aili’	Chromosome	Hap1 584.88 Mb Hap2 578.78 Mb	Hap1 19.84 Mb Hap2 25.17 Mb	Pacbio HiFi Illumina-short Illumina-HiC
‘Baltico’	T2T	Hap1 564.48 Mb Hap2 568.27 Mb	Hap1 37.58 Mb Hap2 38.01 Mb	ONT Ultra long (UL) ONT Illumina-short Illumina-HiC

With the development of sequencing technology and assembly algorithms, the assembly of carnation genomes is becoming more and more perfect. So far, the assembled high-quality carnation genome resources are still relatively poor, and the research on utilizing the genome to mine key genes and apply them to genetic improvement and molecular breeding is not deep enough. The currently released carnation genomes all come from a single individual, restricting their capacity to mirror the species' full genetic diversity and possibly losing crucial genetic variant data. The construction of plant pangenomes offers valuable opportunities to uncover extensive genetic variation, discover novel functional genes, elucidate the molecular basis of variety formation, and enhance the understanding of genetic diversity within species. Pan-genome construction has been attempted in plants such as soybean [18], Arabidopsis [19], cotton [20], tomato [21], potato [22], citrus [23], and apple [24], and significant progress has been made especially in major crops such as rice [25] and maize [26]. So far, *de novo* genomes of 1020 plant species from 13 families have been assembled for pan-genome construction [27].

No species of ornamental plants have yet constructed a pangenome, which is due to the specificity and diversity of ornamental plants, making their genome research a challenge and an opportunity. This is mainly due to the technical challenges of high heterozygosity, repetitive sequences and high ploidy in their genome studies, while morphological diversity increases the difficulty of phenotype–gene association analysis [13, 28]. The construction of carnation pan-genome can analyze genetic variation, excavate functional genes, reveal the mechanism of variety formation, and promote the development of ornamental plant genomics.

A well-developed database is crucial for Carnation multiomics research. Currently, Carnation DB only has scaffold-level data storage and basic analysis functions, which can hardly meet the research needs. Drawing on the experience of genome databases of Arabidopsis [29] and Lotus [30], the construction of a comprehensive carnation database will help integrate genomic, transcriptomic and breeding data, and realize efficient query and in-depth analysis. With the growth of genomic data, the database needs to be upgraded from data storage to online complex analysis to support structural, comparative, and functional genomics research and to facilitate the development of genetic improvement and molecular breeding in carnations.

**Figure 3:**
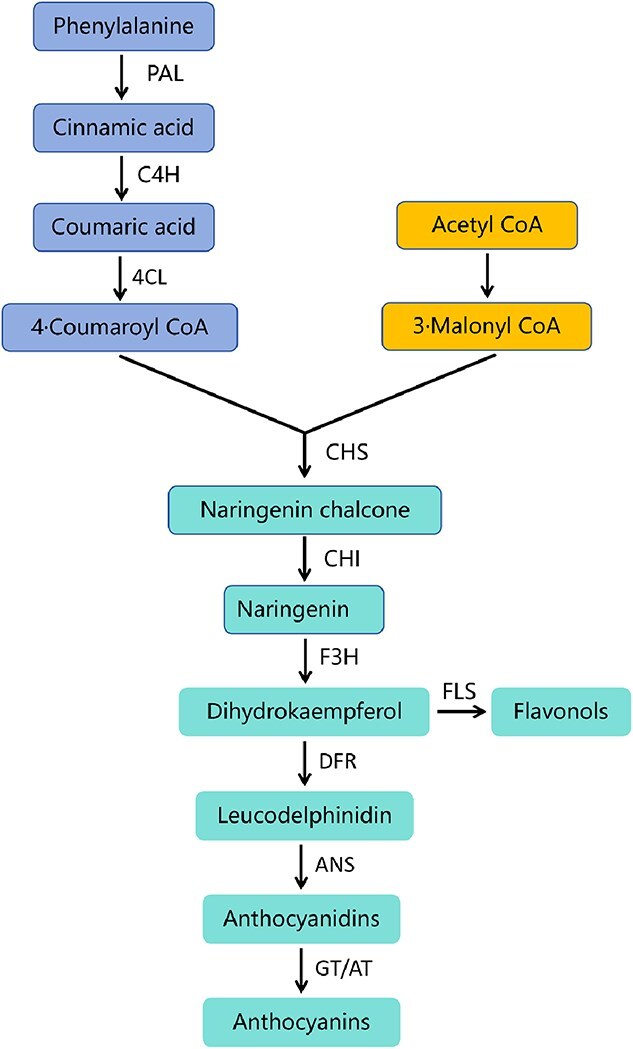
The biosynthesis pathways of anthocyanins and flavonols in carnations. Phenylalanine ammonia-lyase (PAL), cinnamate-4-hydroxylase (C4H), 4-coumarate CoA ligase (4CL), chalcone synthase (CHS), chalcone isomerase (CHI), flavanone 3-hydroxylase(F3H), flavonol synthase (FLS) dihydroflavonol 4-reductase (DFR), anthocyanidin synthase (ANS), acyltransferase (AT), glucosyltransferase (GT).

## The secret of colorful carnation

The diverse range of colors in carnation flower results from the accumulation of anthocyanin and other flavonoids. Most of the genes encoding anthocyanin biosynthesis enzymes have been identified. For example, *DcWRKY15* promotes anthocyanin accumulation by activating the expression of chalcone synthase (DcCHS) and flavanone 3-hydroxylase (DcF3H) [31] (Fig. 3). Carnation petals contain the following four major anthocyanins [32, 33]: pelargonidin 3-malylglucoside (Pg3MG), cyanidin 3-malylglucoside (Cy3MG), pelargonidin 3,5-cyclicmalyldiglucoside (Pg3,5cMdG), and cyanidin 3,5-cyclicmalyl diglucoside (Cy3,5cMdG), giving red, dark red, pink, and purple color, respectively. Notably, the acylation of anthocyanins by malic acid and the formation of cyclic anthocyanin structures by organic acid-binding glycosylation are unique to Dianthus species. In general, one carnation cultivar accumulates only one of the four anthocyanins in the petals as the major anthocyanin [34]. However, wildtype carnations lack a flavonoid 3′,5′-hydroxylase (*F3*′*5*′*H*) gene, preventing the production of delphinidin-type anthocyanins that cause blue color. To overcome this limitation, transgenic violet carnation named ‘Moondust’ and ‘Moonshadow’ were developed by introducing a petunia *F3*′*5*′*H* and dihydroflavonol 4-reductase (DFR) gene into a white carnation lacking DFR gene [35, 36]. Additionally, several carnation cultivars display a dusky and metallic colors caused by anthocyanic vacuolar inclusions (AVIS) [34]. Chalcononaringenin 2′-O-glucoside (Ch2′G) is an important flavonoid pigment that causes the yellow carnation [37], and a part of chalcone to Ch2′G by chalcone 2′-glucosyltransferase, leading to accumulation of Ch2′G and anthocyanin simultaneously to give orange carnation [38]. The appearance of the carnation white flower phenotype is due to the expression of basic helix–loop–helix protein gene [39]. Finally, budding can also lead to color changes. For instance, an active *hAT* transposable element caused carnation bud mutation by inserting *DcF3H* gene, altering the flower color from purple to dark pink [40]. Interestingly, recent research has proved that a few carnation cultivars are able to synthesize and accumulate esterified carotenoids, suggesting that it is possible to produce vivid yellow-flowered carnation by controlling the rate of biosynthesis and esterification of carotenoids in these cultivars [41]. Overall, the color diversity in carnations is a result of complex genetic and biochemical mechanisms involving anthocyanins, flavonoids, and carotenoids, with genetic engineering and natural mutations contributing to novel color phenotypes.

The diversity of flower color patterns is also a vital factor to attract the attention of customers. And these patterns are due to the space-specific regulation of anthocyanin biosynthesis [42]. Recent research has reported 10 different flower patterns in carnations and a ring-like coloration pattern in other *Dianthus* species. It suggests that the pattern with a border where the color density changes gradually might be caused by post-transcriptional gene silencing. On the other hand, the pattern with a border showing abrupt changes in color density may be due to transposon excision [42]. Subsequently, Morimoto collected 110 carnation cultivars and assessed their flower color patterns, it was found that these 110 varieties could be categorized into 5 typical patterns and 2 minor ones. Additionally, the 5 typical patterns were further subclassified into 16 subtypes [43]. In parallel, combined with carnation transcriptome analysis, genes such as *MYBs*, *bHLHs*, and *WRKY44* were screened. These genes were found to act cooperatively on anthocyanidin synthase (ANS), playing a crucial role in regulating carnation multicolor formation [16]. And *DcBHLH1/2* participates in the formation of white edge of carnation petals by inhibiting the synthesis of anthocyanins [44].

At present, the molecular mechanism of pure color carnation has been studied to some extent: white [45], pink [46], and yellow [47], but it is still not comprehensive, and the potential molecular mechanism of multicolor flowers is not clear, further research is needed to provide direction for the breeding of novel carnation cultivars.

## The vanishing scent of carnation

Carnation fragrance includes benzenoids/phenylpropanoids, terpenoids, fatty acid derivatives, and other minor components [48, 49]. Particularly, the floral volatile benzenoids/phenylpropanoids (FVBPs) are the principal components [48]. Most carnation cultivars have a fruity scent caused mainly by benzenoid methyl benzoate. Additionally, a few cultivars also possess a spicy and clove-like odor based on eugenol benzenoid [48], which have a comparatively long duration of obvious scent. However, the carnation scents disappeared rapidly after harvest [50], with a 15%–50% reduction in scent emissions from cut flowers within 2 days after harvest. It is expected that the duration of a noticeable scent in carnation cut flowers will be extended by wet transport rather than dry transport [51].

In the past, oils from fragrant carnation flowers were used as brilliantines, hair oils, talcum powders, and soaps in southern Europe and North Africa [52]. However, the variation and intensity of fragrant carnation have been lost because benzenoid levels are markedly lower in modern carnation [48]. Structural variations in exons of carnation eugenol synthase (*EGS*) gene may be responsible for carnation eugenol loss [16]. On the other hand, from a natural perspective, one of the key functions of flower scent is to attract insects to pollinate [53]. The disappearance of floral fragrance may also be due to its gradual abortion. Wild Dianthus species, especially rich in methyl salicylate, *β*-oxylenes, and *β*-caryophyllene, have a pleasant smell and is an important genetic resource for introducing fragrance into carnations [54]. Most hybrids derived from crossing wild Dianthus species with carnations exhibit an increased diversity and quantity of scent compounds compared with parental carnations, confirming the effectiveness of interspecific hybridization in improving flower scent [55]. However, interspecific hybridization is a time-consuming process, so it needs to study the molecular mechanism of carnation flower aroma synthesis and try to shorten the breeding time by gene editing technology.

## The riddle of carnation double flowers formation

Flower morphology is an important breeding target of carnation, and the double-flowered carnation attracts more attention. Saunders [56] firstly reported that the floral phenotype of carnation was a monogenic trait and named the locus involved ‘*D*’. Subsequently, Onozaki *et al.* [57] identified the single carnation flower allele ‘*d*’ derived from *D capitatus* ssp. *andrzejowskianus*. Yagi *et al.* [58] identified that the *D_85_* locus controlling flower phenotype and two SSR (CES0212 and CES1982) were tightly linked to the *D*_85_ locus. More recently, Wang *et al.* [59] suggested that the C-class (*DcaAG*) gene might play a significant role in the carnation double flower and the ectopic expression of class A and class C genes in carnation may be an important factor affecting the formation of double-flowered carnation. Although carnation double flower is an important ornamental trait, the molecular mechanism of carnation double flower formation is still limited and needs to be further explored.

## The stress resistance of carnation

Fusarium wilt and bacterial wilt are the two main diseases causing serious economic damage to carnation industry. *Fusarium oxysporum* f. sp. *Dianthi* (*Fod*) is the pathogen of vascular wilt of carnation. To combat this disease, recent studies have shown promising results with the use of biological elicitors. For instance, the addition of biological elicitor (i.e. ultrasound-assisted dispersion obtained from *Fod* mycelium) to soil can be used as a resistance inducer to reduce the occurrence of fusarium wilt [60]. Additionally, thiamine treatment before *Fod* inoculation could activate early defense mechanisms and promote the accumulation of specific phenolic compounds in carnations, enhancing resistance against vascular wilt [61, 62]. As for bacterial wilt, Yagi *et al.* introduced the first bacterial wilt-resistant carnation variety ‘Karen Rouge’ in 2010 and identified line 85–11 in 2012, which showed significant resistance to bacterial wilt. To further understand and utilize this resistance, an SSR-based genetic linkage map was constructed using the F2 population of lines 85–11 and susceptible variety ‘Pretty Favvare’. This map identified markers closely linked to bacterial wilt resistance and can be directly used in molecular marker-assisted selection (MAS) to facilitate the introduction of target resistance genes into some cultivars [63]. In addition, carnation’s common viruses are carnation mottle virus (CarMV), carnation etched ring virus (CERV), carnation latent virus (CLV), carnation necrotic fleek virus (CNFV), carnation Italian ringspot virus (CIRV), carnation cryptic virus 3 (CCV3-IR1 isolate), and so on. The CLV complete genome sequence and CCV3-IR1 were reported by high-throughput sequencing (HTS) [64, 65]. CarMV is a single positive-strand RNA virus, and using transcriptome data, Yeonhwa Jo *et al.* firstly provided a nearly complete genome sequence of carnation mottle virus (CarMV) infecting hop plants [66]. Recently, Breit *et al.* reported the genomic sequences of 10 CarMV variants, all of which showed nucleotide differences, but four of which did not show any variation at the amino acid level [67].

Carnation occupies an important position in the global flower industry, but its sensitivity to high temperature seriously affects its growth and quality [68]. In order to improve the heat tolerance of carnation, researchers have explored its response mechanism under high-temperature conditions through various studies [69]. Heat shock proteins (Hsps), as key molecular chaperones, are involved in protein folding and protection, and are essential for plants to cope with heat stress. The results showed that the expression of Hsp20 and Hsp90 family members in carnation was significantly up-regulated under high temperature, and the effect of DcHsp17.8 on heat tolerance was verified in transgenic Arabidopsis, the implementation process involves enhancement of antioxidant enzyme activity and mitigation of cell membrane damage [70, 71]. Meanwhile, the heat shock transcription factors (Hsfs) gene family in carnation has also been comprehensively identified [72], in which the *DcHsfA1d* gene plays a positive regulatory role in the response to high-temperature stress [73]. In addition, the NAC transcription factor family plays an important role in regulating plant heat tolerance. In particular, *DcNAC41* is significantly up-regulated by high temperature. Its heterologous expression in *Arabidopsis thaliana* significantly enhances the heat tolerance of plants, including increasing antioxidant enzyme activities, reducing reactive oxygen species (ROS) accumulation, and enhancing photosynthetic efficiency. Meanwhile, some NAC family members finely regulate the heat tolerance response of plants by regulating ROS homeostasis and the expression of heat shock proteins [74]. Finally, the researchers found that melatonin use could improve carnation heat tolerance by upregulating the transcription of stress-related genes [75, 76]. These findings provide a basis for further understanding the heat tolerance mechanism of carnation, and provide a new strategy for breeding heat-tolerant carnation varieties, which is expected to improve their adaptability to high-temperature environments without sacrificing the ornamental quality of carnations.

## Carnation for long vase life

The vase life of carnation cut flowers is a substantial limitation in the market and affect its ornamental value. In general, early post-harvest senescence is caused by the production of ethylene [77]. After harvest, the petals roll and bacteria accumulate on the cut surface of the stem, producing exopolysaccharides that block xylem vessels and thus increase hydraulic resistance, this subsequently leads to reduced water uptake by the stem and premature withering [78].

In the past few years, scientists have mainly used hybridization to obtain long-lived carnation varieties [79]. In recent years, with the rise of genetic engineering and gene editing technologies, researchers have begun to genetically analyze the aging of cut carnation flowers, and are trying to extend its vase life with environmentally friendly preservatives.

## Ethylene-induced carnation petal senescence

Carnation is a highly ethylene-sensitive flower, and its senescence is characterized by self-catalytic ethylene production, followed by petals withering. During carnation natural senescence, a large amount of ethylene is produced first in the pistil and then in the petals, increased ethylene production accelerates the curling of petals, leading to wilting, so carnation is often used as a model plant to study ethylene-induced petal senescence [15, 80, 81]. Moreover, ethylene can promote the release of endogenous ethylene in the process of petal senescence, thus promoting the senescence of cut flowers. Therefore, ethylene is an important determinant of carnation vase life.

As early as 1864, there was a record of several trees being damaged by an illuminating gas leaked from pipes. The carnation growers also found that the gas seeped from the defective pipes through the ground into greenhouses caused carnation heavy loss, when the pipes were repaired, the injuries ceased. It was later proved that the gas, which caused damage to trees and carnations was ethylene, and carnations are sensitive to relatively low concentrations of ethylene [77]. In 1967, ethylene was recognized as a hormone involved in regulating flower senescence [82].

Today, ethylene biosynthesis and signal transduction pathways have been well studied, related genes have also been cloned and identified [83–92]. Ethylene biosynthesis is the conversion of methionine (Met) to S-adenosylmethionine (SAM), then 1-aminocyclopropane-1-carboxylic acid (ACC) synthase (ACS) catalyzes SAM to ACC, which is a rate-limiting step in ethylene biosynthesis, the conversion of ACC to ethylene is oxidized by ACC oxidase (ACO) [93]. In the ethylene signaling transduction pathway, ethylene is perceived by a family of receptors located on the endoplasmic reticulum (ER) membrane. In Arabidopsis, these receptors include ethylene response1/2 (ETR1/2), ethylene response sensor (ERS1/2), and ethylene insensitive4 (EIN4), resulting in inactivation of the constitutive triple response1 (CTR1) complex [94–96]. This inactivation prevents the phosphorylation of ethylene insensitive2 (EIN2). As a result, EIN2 is not degraded by F-box proteins EIN2-targeting protein 1 and 2 (ETP1/2) and undergoes spontaneous cleavage. The cleaved C-terminal domain of EIN2 (EIN2-C) is then transported to the nucleus [97]. Once in the nucleus, EIN2-C activates the downstream transcription factors EIN3 and EIN3-like1 (EIL1). These transcription factors bind to the promoters of ethylene response factors (ERFs), which are plant-specific transcription factors involved in modulating downstream gene expression in response to ethylene and environmental stresses [97, 98]. Despite significant progress in understanding ethylene biosynthesis and signaling, the underlying mechanisms of its regulatory network, particularly in specific contexts such as flower senescence, remain incompletely understood.

**Figure 4:**
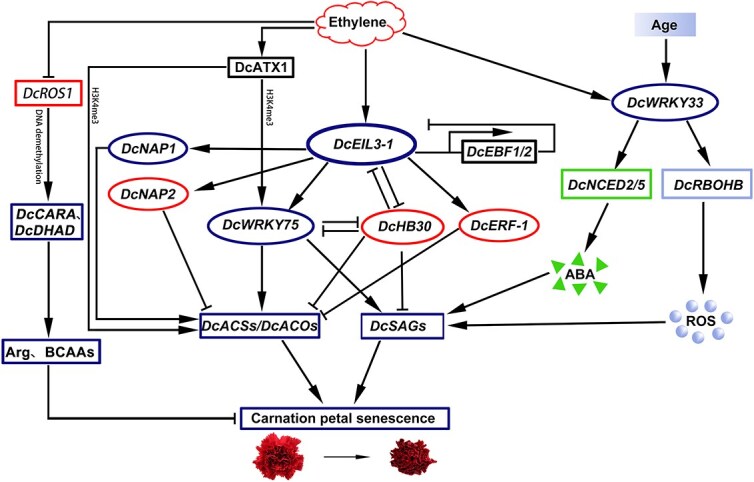
Molecular mechanism of ethylene-induced petal senescence in carnation. EIL3–1: Ethylene-insensitive3-like 1; ATX1: Arabidopsis homolog of trithorax1; HB: Homeobox; NAP: NAC-Like, Activated by AP3/PI；CARA: Carbamoyl-phosphate synthase subunit A; DHAD: Dihydroxyacid dehydratase; ROS1: Repressor of silencing1; BCAAs: Branch chain amino acids; Arg: arginine; NCED: Nine-cis-epoxycarotenoid dioxy genases; RBOHB: respiratory burst oxidase homolog.

To further explore how ethylene induces the senescence of carnation petals, we performed transcriptome high-throughput sequencing on the petals of the ethylene-sensitive carnation cultivar ‘Master’ [99]. Through differentially expressed genes (DEG) analysis and weighted gene co-expression network analysis (WGCNA), four core transcription factors were mapped: three from the WRKY family and one from the NAC family, which play pivotal roles in ethylene-induced petal senescence. *DcWRKY75*, is a direct target gene of the ethylene signaling master regulator DcEIL3-1, binds to the promoters of *DcACS1*, *DcACO1,* and *DcSAGs* (senescence-associated genes), thereby promoting petal senescence [100]. This highlights the direct involvement of WRKY transcription factors in mediating ethylene responses through transcriptional regulation of downstream effectors. Another notable finding is the antagonistic relationship between DcWRKY75 and DcHB30, which suggests a fine-tuned regulatory mechanism where these transcription factors counterbalance each other’s effects on petal senescence [101]. Additionally, the mutual repression between DcEIL3-1 and DcHB30 further underscores the complexity of transcriptional feedback loops within the ethylene signaling pathway [102]. We also revealed that DcERF-1, an ethylene response factor, negatively regulates ethylene biosynthesis by inhibiting the expression of *DcACO4*, indicating a potential feedback mechanism to modulate ethylene levels [103]. Moreover, DcWRKY33 was shown to participate in ABA and ROS-induced senescence by binding to the promoters of ABA biosynthesis genes (*DcNCED2*, *DcNCED5*) and an ROS-generating gene (*DcRBOHB*) [104]. This crosstalk between ethylene, ABA, and ROS pathways highlights the multifaceted nature of petal senescence regulation. In the NAC family, DcNAP1 and DcNAP2 were identified as ethylene-induced transcription factors with opposing roles in petal aging. DcNAP1 accelerates aging by activating ethylene biosynthesis genes, while DcNAP2, which contains a transposon-induced domain deletion, interferes with DcNAP1’s activity to delay aging. This ‘gas and brake’ mechanism provides a unique perspective on how genetic variations can influence transcription factor function and downstream outcomes [105]. Finally, in the presence of ethylene, DcEIL3-1 can activate the expression of *DcEBF1*/*2*, which can target DcEIL3-1 protein degradation through ubiquitination and 26S proteasome system, thereby negatively regulating ethylene signal transduction [106]. This feedback loop ensures that ethylene responses are tightly controlled and transient.

In recent years, many studies have revealed that epigenetic regulation is involved in ethylene signal transduction [107–112]. So, we investigated whether epigenetics affect the senescence of carnation petals via the ethylene signaling pathway. DcATX1, a histone H3K4 methyltransferase, was shown to regulate the transcription of *DcWRKY75*, *DcACO1*, *DcSAG12,* and other potential downstream target genes, thereby affecting the senescence of carnation petals [113]. But how DcATX1, which has no recognition function, binds to the promoter of corresponding downstream genes is still unclear and needs further study. DNA methylation was also implicated in the regulation of flower senescence, with the DNA demethylase repressor of silencing1 (DcROS1) regulating DNA promoter hypermethylation of important amino acid biosynthesis genes (*DcCARA* and *DcDHAD)*, they regulate flower senescence by controlling amino acid content [114] (Fig. 4). We also extended research to potted carnations, which are less sensitive to ethylene compared to cut carnations. Water stress and endogenous ethylene release were identified as the main factors determining flower longevity, while exogenous ethylene has little effect on the volatiles of potted carnations [115]. This suggests that the regulatory mechanisms of petal senescence may be different in cut and potted carnations, with the latter relying more on intrinsic factors than external ethylene exposure.

The comprehensive investigation of ethylene-induced petal senescence in carnations provides valuable insights into the intricate regulatory network involving transcription factors, epigenetic modifications, and crosstalk with other signaling pathways. The findings not only advance our understanding of flower aging, but also offer potential targets for manipulating ethylene responses in horticultural crops. Future research should focus on elucidating the molecular mechanisms underlying the binding of epigenetic regulators to gene promoters and exploring the differences in ethylene sensitivity between cut and potted carnations. This knowledge could be instrumental in developing strategies to enhance flower longevity and improve post-harvest quality.

## Breeding for long vase life carnation varieties

Onozaki *et al.* firstly selected six commercial standard carnation cultivars with wide variation in vase life as their initial breeding materials: four Mediterranean-type cultivars (‘Pallas’, ‘Sandrosa’, ‘Candy’, and ‘Tanga’) and two Sim-type cultivars (‘White Sim’ and ‘Scania’). After seven generations of hybridization, the average vase life increased from 7.9 days to 15.9 days, a net increase of 8.5 days [116, 117], which shows that the selection from the first generation to the sixth generation is effective. At the same time, they have produced several long vase life and low-ethylene-sensitivity cultivar, such as ‘Miracle Rouge’ and ‘Miracle Symphony’ and ‘806-46b’, among others, line ‘806-46b’ has a vase life of 27.1 days and low ethylene sensitivity [79]. However, it takes a long time to get the long vase life cultivar through traditional cross breeding, understanding the molecular mechanism of carnation senescence will help to shorten the breeding period through genetic engineering.

## Genetic transformation of carnation

The cut flower market is currently in need of carnation varieties with superior traits. However, the traditional breeding methods with long breeding years have failed to keep up with the market demand. In contrast, genetic transformation technology has gained significant attention from researchers due to its numerous advantages. This technology not only dramatically shortens the breeding cycle for new varieties but also allows for targeted modification of specific traits without being restricted by the genetic limitations of parental lines. Therefore, the development of efficient genetic transformation technology is of great significance for the molecular breeding and functional gene research of carnation.

The establishment of carnation genetic transformation system primarily involves the use of organs such as leaves, stem segments, cotyledons, lateral branches to develop a stable transformation receptor system. The transfer of target gene is achieved by *Agrobacterium tumefaciens* infection of explants. This method is widely used due to its relatively high-transformation efficiency, clear insertion fragments, and good genetic stability. In addition to agrobacterium-mediated transformation, transient expression of target gene in carnation has also successfully achieved by PEG-mediated methods [118, 119]. Despite these advancements, the current carnation genetic transformation system still faces several challenges, including complex operational procedures, low transformation efficiency, and difficulties in reproducibility. To address these issues, researchers have implemented various optimization strategies. Such as the use of plant growth regulators (PGRs) is a proven approach, with auxins promoting rooting [120] and cytokinins enhancing the proliferation of callus tissues [121]. By finely tuning the concentrations and ratios of different PGRs, the transformation efficiency and regeneration capacity of explants can be significantly improved. In additionally, the expression of developmental regulators (DRs) [122–124] has been explored to regulate the development-related genes of carnations, thereby enhancing the efficiency of genetic transformation. Meanwhile, acetosyringone [125] is used to induce the expression of Vir genes in *Agrobacterium*, enhancing its transformation ability and antioxidants [126] are applied to mitigate oxidative damage to explants, improving their survival rate and overall transformation efficiency.

On the other hand, CRISPR-Cas9 technology, as a novel third-generation artificial endonuclease, has many advantages such as high efficiency, simple operation and low cost [127, 128], which shows a very broad application prospect. At present, CRISPR-Cas9 technology can complete gene editing activities such as Gene Knockout [129], base editing [130], gene repression or activation [131], and multigene editing [132] in plants. In 2024, researchers successfully applied CRISPR-Cas9 technology to the genetic transformation of carnation for the first time, targeting the ethylene biosynthesis gene [133] and the anthocyanin synthase (ANS) genes [134]. The study demonstrated that single-stranded guide RNAs (sgRNAs) could specifically cut these target genes, resulting in successful edits. However, the multistep electroporation method used, while capable of inducing mutations, had limited effects on flower color. This suggests that further optimization is needed to enhance mutation efficiency and achieve more pronounced phenotypic changes. To address these challenges, researchers developed a gene-editing system using seed-propagated carnation leaves as experimental material. They achieved an editing efficiency of 6.7% for the phytoene desaturase (PDS) gene, successfully inducing albino buds through optimized medium formulations and antibiotics [135]. Despite these advancements, the application of this system is constrained by the heterozygous nature of most commercial carnation varieties, which are propagated asexually to avoid trait segregation. This limits the widespread use and effectiveness of the current editing system. Another critical factor in optimizing CRISPR-Cas9 efficiency is the choice of promoter. Researchers identified the DcU6 promoter from carnation and demonstrated that it significantly enhances editing efficiency compared to the AtU6–29 promoter when driving sgRNA expression in protoplasts [136]. This finding highlights the importance of promoter selection in improving gene-editing outcomes and lays a foundation for future improvements in carnation genetic transformation.

Finally, in addition to the traditional transgenic technology, carnation’s genetic transformation can also learn from other species emerging simple and efficient transformation methods. For example, the regenerative activity-dependent in planta injection delivery (RAPID) [137], in which *Agrobacterium tumefaciens* is injected directly into the base node of the sweet potato stem, and further improved on the ‘Cut-dip-bud’ (CDB) method, direct infection of the CDB gene delivery system [138], which cleaves succulent plant leaves, is a simple and efficient method of genetic transformation based on the plant’s ability to regenerate autonomously and to reproduce asexually, without the need for tissue culture. In addition, gene transformation can also be achieved by *Agrobacterium tumefaciens* for induction of meristems in *Bacillaria tabacum* grown in sterile culture or soil by delivery of WUS2 and BBM or other developmental regulators (DRs) [139]. These methods are simple, time-consuming, and do not need tissue culture, it is worth trying in carnation.

In short, the carnation genetic transformation system needs a variety of methods to continue to explore. We should not only continue to optimize the experimental conditions on the basis of previous research, and actively learn from the successful experience of other plant transformation system, to improve the transfer efficiency of the target gene and the regeneration efficiency of the transformed receptor. It is necessary to combine with genome editing technology such as CRISPR and use molecular technology to achieve more efficient character improvement. It contributes to carnation breeding and germplasm resources innovation.

## Conclusions and perspectives

Carnation is one of the most important flower crops in the world. The carnation’s color, shape, fragrance and vase life directly affect whether consumers buy carnations again, and the disease resistance of carnations determines whether carnations can be planted by producers in large quantities, thus producing economic benefits. However, there are few literature on carnation for a full-scale interpretation. In this review, we made a comprehensive summary of carnation’s flower color, fragrance, flower shape, disease resistance, and vase life. It is expected to provide guidance for the future carnation industry.

With the success of carnation genome sequencing in 2014, a new era of carnation molecular world has officially begun. The transcriptome sequencing results of carnation have also been published, the major carnation gene families have been identified one after another, and the molecular mechanisms of disease resistance and vase life have been deeply studied, but little is known about the protein modification and its function of carnation, such as phosphorylation, ubiquitination, glycosylation, etc., if possible, proteomic and single-cell sequencing of carnation can be carried out to further explore the potential conservation genes and modifications of carnation, which will help the breeding and industry of carnation later.

Historically, carnations were fragrant and used in spices, soaps, and essences, but modern varieties have lost this original fragrance. So where did this missing scent go? It may have been gradually lost over the 2000-year breeding history of carnations. Can this lost scent be recovered? It is expected that the molecular mechanism of carnation evolution will be elucidated and the lost aroma will be found by collecting carnation germplasm resources and integrating various omics methods.

In recent years, as people’s economic level and cultural demand have risen, flowers have gradually become a part of people’s daily life. Among them, carnations have gained immense popularity for their diverse colors, exquisite flower patterns and beautiful implication of maternal love. They are not only favored on Mother’s Day, but also widely used on Women’s Day, Chinese Valentine’s Day and numerous other festivals as a way to celebrate women. These occasions offer opportunities to honor women. Therefore, carnations can becalled ‘the ladies flower’, which stand as a floral representation of women, lauding every woman for her unwavering courage and inherent beauty.
